# Schizophrenic with childhood trauma: characterization of a Tunisian sample

**DOI:** 10.1192/j.eurpsy.2023.2206

**Published:** 2023-07-19

**Authors:** A. Syrine, F. Rim, B. Olfa, G. Imen, S. Najeh, O. Sana, M. B. Manel, Z. Lobna, C. Nada, B. T. Jihen, M. Mohamed

**Affiliations:** Psychiatry ‘C’ Department, University Hospital of Hedi Chaker, Sfax, Tunisia

## Abstract

**Introduction:**

Schizophrenia is a neurodevelopmental process affecting approximately 1% of the population. Multiple studies have found that Childhood trauma is an important risk factor in the emergence and clinical course of schizophrenia.

**Objectives:**

The purpose of this study was to assess the characteristics of schizophrenic inpatients with childhood trauma among a tunisian sample.

**Methods:**

Stabilized inpatients with schizophrenia at the Psychiatry C department at University Hospital in Sfax were involved in our study. Sociodemographic and clinical data of patients were collected from medical records.

We used Childhood Trauma Questionnaire-Short Form (CTQ-SF) to evaluate the occurrence of childhood maltreatment.

**Results:**

We recruited 33 patients, all men with a mean age of 35 years and 4 months (SD=9.44 years).

They were married in 6.1% and 24.2% of patients had regular work.

The mean age of onset of the disorder was 24.42(3.25).The level of poor psychotropic medication adherence was 72.7%.

According to CTQ-SF, 78.8% of patients had experienced child trauma with a mean score of 35.67 (SD =8.61).

A rate of 39.4% reported having experienced one child trauma, while 60.6% reported having experienced more than one.

We found high rates of emotional neglect (87.8%) while emotional and physical abuse during childhood were experienced by 39.4%, and 6% respectively and physical neglect were found in 30.3% of cases.

Patients with more than one childhood trauma were found to have an earlier onset of psychosis (p=0.004)

The occurrence of childhood trauma was not associated with the socio-demographic characteristics of the respondents or the clinical features of the disease.

**Conclusions:**

The results point toward childhood emotional neglect being of specific importance to schizophrenia, which may be an area for future prevention and clinical attention.

**Disclosure of Interest:**

None Declared

